# Gut health modulation through phytogenics in poultry: mechanisms, benefits, and applications

**DOI:** 10.3389/fvets.2025.1616734

**Published:** 2025-08-20

**Authors:** Aderanti Ifeoluwa Oni, Oyegunle Emmanuel Oke

**Affiliations:** Department of Animal Physiology, Federal University of Agriculture, Abeokuta, Nigeria

**Keywords:** gastrointestinal tract, phytobiotics, avians, health, productivity, microbiota

## Abstract

The potential of phytogenic feed additives (PFAs) to modulate gut health has drawn much attention as natural alternatives to antibiotics in poultry production. These plant-derived compounds, such as polyphenols, alkaloids, flavonoids, and essential oils exhibit various bioactive properties that improve gut microbiota composition, support immune function, and improve nutrient absorption by influencing gut morphology and digestive enzyme activity. Their antioxidant, anti-inflammatory, and antimicrobial properties help maintain and improve overall poultry performance and lower the prevalence of diseases related to gut and intestinal integrity. By promoting a balanced gut microbiota, phytogenics help reduce enteric infections, promote beneficial bacteria, and suppress pathogenic microbes, reducing the need for antibiotic growth promoters. PFAs are valuable tools for sustainable poultry production since they have been connected to enhanced feed conversion efficiency, growth performance, and meat quality in addition to their health benefits. However, further research and standardization are needed to address issues, including regulatory compliance, appropriate dosage determination, and variability in the composition of bioactive compounds. To improve their stability and effectiveness, future research should improve PFA formulations, examine their synergistic interactions with other feed additives, and create innovative delivery systems such as microencapsulation. A promising strategy for enhancing animal welfare, lessening the impact on the environment, and guaranteeing antibiotic-free poultry production is the incorporation of phytogenics into poultry feed. Phytogenics can play a significant role in the future of sustainable poultry farming by tackling the current issues and maximizing their uses.

## Introduction

One of the biggest food sectors worldwide is the poultry sector (1). Due to the need for protein sources to feed the world’s expanding population, this industry is rapidly expanding within the meat-producing agricultural sector (2, 3). It is anticipated to reach 121% of 2005 production by 2050 (4). In many regions of the world, it is constantly growing and becoming more industrialized (5). The production of broilers, in particular, has demonstrated rapid growth in both worldwide meat demand and business profit, both of which will increase over the next century (6). The modern broiler is distinguished by its rapid growth and extremely effective feed conversion. As such, performance necessitates a large feed intake, and the gastrointestinal (GI) tract’s physiology is under tremendous strain (7–9), especially in the face of the changing climate (10). The optimal performance of poultry greatly depends on a healthy intestine. Damage to the gut reduces the body’s ability to use nutrients, impacting birds’ health and development (11).

One crucial organ that mediates the intake and utilization of nutrients by birds is the gastrointestinal tract (GIT). The GIT is regarded to be the biggest organ in the immune system because over 70% of its cells can call it home (12). It serves as a crucial interface between the host and its surroundings, facilitating proper immune responses, tolerance to benign and beneficial microorganisms, and the digestion and absorption of nutrients. It also stops intestinal microorganisms from moving to sterile areas (13).

The breakdown of complex foods, which improves the absorption of essential vitamins, minerals, and amino acids, is largely dependent on a healthy gut microbiota. An effective digestive system in poultry can result in increased feed conversion ratios (FCR), better weight gain, and enhanced production performance. The nutrients consumed can have a major impact on the growth and operation of the GIT. Also, diet composition (ingredients, nutrients, and additives) can affect the formation and function of the digestive system, including the immune system and the microbiota (14). Improved enzyme activity and digestive secretions are significant elements that impact gut function and enhance nutrition digestion (15).

For the poultry industry, gut health maintenance is essential. Poultry’s immune system, intestinal integrity, gut microbiota, and diet interact continuously to influence gut health and function, affecting poultry’s performance. Gut health consists of intestinal antioxidant function, morphology barrier function, immune response and gut microbiota modulation, and they all work together to promote gut homeostasis for improved performance and health in animals.

The gut performs a number of tasks, such as absorbing nutrients, absorbing and secreting water and electrolytes, secreting mucin and immunoglobulins, and providing a selective barrier that protects against pathogens and dangerous antigens (16). The idea that the gut actively contributes to organ integrity and body defence along with its absorption, secretion, and barrier functions has surfaced in the last 10 years (17, 18).

Phytobiotics, another name for phytogenic feed additives (PFAs), are a broad class of bioactive substances produced from plants that are utilized in animal nutrition to support productivity and health. PFAs include a wide range of compounds that are extracted from different plant components, such as seeds, roots, leaves, and flowers, such as spices, oleoresins, herbs and essential oils (19–21). The growing interest in PFAs is largely driven by the global shift toward natural alternatives in poultry production due to concerns about antimicrobial resistance. In poultry, phytogenics provide a variety of biological actions that enhance gut health and overall performance, such as anti-inflammatory, antioxidant, antibacterial, immunomodulatory, and digestive-enhancing properties (22, 23). As interest in sustainable livestock production grows, phytogenics have emerged as potential candidates for improving feed efficiency, lowering the environmental impact of poultry farming, and improving gut health. Factors such as the animal’s physiological state, processing methods, dosage, and botanical origin affect these compounds’ effectiveness. This review examines the role of phytogenics in modulating gut health in poultry production, emphasizing their methods of action, possible advantages, and valuable uses. By synthesizing current research findings, it seeks to provide insights into how phytogenics can improve overall performance, immune response, microbial balance, and intestinal integrity, thereby offering sustainable alternatives to conventional growth promoters.

### Gut microbiota

In recent years, the significance of intestinal microbiota in poultry nutrition, health, physiology, and immunity has emerged (24). The commensal microbiota, affixed to the intestinal epithelium, is crucial for preserving homeostasis and preventing pathogen colonization (25). In order to guarantee that commensal microorganisms are tolerated, pathogens are identified and controlled, and potentially harmful commensal microorganisms like *Clostridium perfringens* and *Escherichia coli* are kept in check, the microbiota works in tandem with the chicken immune system, aiding in the immune cells’ maturation and training (26). Additionally, it increases the epithelium absorptive surface by producing metabolites, including SCFAs, indoles, vitamins, and antimicrobial compounds, which aid in the formation of the lamina propria, mucus layer, and epithelial monolayer (27).

The gut microbiota is the first line of defence against infections and significantly affects chickens’ nutritional, physiological, and health conditions (28). The gut microbiota has been divided into pathogenic (*E. coli*, Campylobacter, and Salmonella) and beneficial (Lactobacilli and Bifidobacteria) bacteria based on their effects on the host (29). Competition for resources, the generation of harmful substances (such as volatile fatty acids and low pH), competition for binding sites on the gut epithelium, and immune system stimulation are some of the ways that the gut microbiota inhibits pathogens (169).

Poultry’s early gut health, gut microbiota, nutritional status, and immunological status have been reported to greatly impact their growth and development (30, 31). Dietary components can affect health and growth performance during post-hatch life by altering the gut microbiota and the intestinal production of metabolites (32). According to Bindari and Gerber (27), the gut-health interaction is the symbiotic equilibrium between the intestinal tract and the microbiota, which means that animal welfare and health are unaffected and are regarded as significant determinants. The immune system and general health are also impacted by the gut flora (33). Human antibiotic resistance and infection can also be determined by the microbiota of the chicken digestive tract (34). The digestive tract indicates the habitat of the microbiota that influences chickens’ digestive systems. Essential biological processes like human physiological ageing, dairy cow methane emission, pig nutrition digestion, absorption and metabolism, and chicken health and productivity are all significantly influenced by the gut microbiota (35, 36).

A key factor in regulating the activation and control of the immune system in broiler chickens is the microbiota in the digestive tract, which varies depending on the feed, location, and age of the bird (37). In the early stages of their development, newly hatched chicks depend on innate immune responses. Microbial administration is one method to increase and stimulate the immunological development of chickens both before and after hatching (38).

There are several ways in which the microbiota’s function in the host’s physiological, nutritional, developmental and immunological processes benefits the gut health, productivity, and general wellbeing of chickens (37). In addition to helping with digestion and nutrient absorption, the gut microbiota also supports the development and operation of the host immune system, the intestinal epithelial barrier, and the competition with pathogenic microorganisms to stop their detrimental spread (39). Intestinal health is largely determined by the symbiotic connection between the host and gut microbiota under normal circumstances. However, “dysbiosis,” or an unbalanced host-microbe connection, can result from a disruption in the gut microbiota (40).

### Challenges to gut health

Poultry welfare and gut health are closely related since poor gut health can result in lower feed intake, growth performance issues, and digestive diseases. The severity of intestinal damage linked to coccidiosis, necrotis enteritis, and general intestinal inflammatory and disease disorders including dysbiosis has been assessed using gut health score systems (15, 27).

The term “dysbiosis” describes an imbalance or disturbance in the gut microbiota in which harmful bacteria multiply and helpful microbes decline. Poor nutrition, stress, antibiotics, and other environmental variables can all lead to dysbiosis. Dysbiosis in chickens can have a number of detrimental effects. By harming the intestinal lining and decreasing the availability of enzymes that aid in food digestion, dysbiosis might hinder the gut’s capacity to absorb vital nutrients (41). This results in lower feed conversion efficiency (FCR) and subpar growth performance.

Dysbiosis can also result from factors that upset the balance of the gut microbiota, such as transportation, changes in the environment, or crowding. The gut’s capacity to maintain a healthy barrier function may be compromised by this imbalance, raising the possibility of inflammatory reactions and pathogen invasion. Additionally, gut motility and feed conversion efficiency can be impacted by chronic stress (42). The gut’s immune system is weakened by dysbiosis, which also lowers the generation of antimicrobial peptides. This increases the risk of gastrointestinal diseases such as coccidiosis and necrotic enteritis.

A number of risk-reduction techniques have been investigated in light of the dangers posed by gastrointestinal disorders, dysbiosis, and antibiotic resistance; one such strategy is using feed additives.

### Feed additives

Understanding the microbial communities in the gut is important for creating safe feed additives and modifying diets to improve gut health and performance (15). Feed additives are natural, non-nutritive ingredients added to the basic diet in small amounts to enhance the quality of feed and animal-based foods and the health and performance of animals. They enhance animal development, intake, absorption, and nutritional assimilation by influencing physiological processes like stress resistance and immunological function (19, 43).

Poultry nutritionists became interested in natural feed additives replacing AGP products (3, 44, 45). Among the many natural feed additives for chicken feed, phytogenic feed additives (PFAs) are highly recommended and accepted by consumers (46–49).

### Phytogenics and gut health

PFAs are naturally occurring, are diverse groupings that are less harmful, leave no residue, and are perfect non-antibiotic growth promoters. They are made from plants, herbs, fruit, spices, and their essential oil, and they are utilized as feed additives in the production of meat animals (43, 45). PFAs are a diverse class of bioactive plant-derived compounds or plant-based products high in phenolics and flavonoids added to animal meals to increase productivity (20, 50). Phytogenic compounds are a wide range of active ingredients that can be obtained from different types of plants. Categories of phytogenic compounds include oleoresins, spices, botanicals, herbs, and essential oils.

Phytogenics contribute to better gut health through their capacity to enhance the gut microbiota, facilitate digestion, and increase immunity. They can have a favorable impact on the gut microbiota by encouraging the growth of beneficial bacteria and preventing the formation of pathogenic microbes. A more diversified and balanced microbiota results from this, which is crucial for preserving gut health and averting gastrointestinal disorders (51). Through the stimulation of digestive enzyme release, increased nutrient absorption, and enhanced enzyme activity, phytogenics can increase the efficiency of poultry digestion. Growth, feed consumption, and general productivity all improve as a result. The digestion of carbs, lipids, and proteins is facilitated by digestive enzymes, including amylase, lipase, and protease, which are secreted in response to phytogenic stimulation. This aids in the more effective digestion and absorption of nutrients by poultry, especially in young birds or stressed birds (52).

Through tight junction protein enhancement and intestinal epithelial integrity promotion, phytogenics can fortify the gut barrier and avert leaky gut syndrome. A stronger gut barrier lowers the risk of systemic infections and enhances general health by preventing toxic substances and dangerous microorganisms from entering the bloodstream. Liu et al. (29).

Given their beneficial impacts on animal health, phytogenics are becoming increasingly popular as affordable feed additives. PFAs can be added to the diet in dry form or as extracts, and the makeup of the bioactive biomolecules in them differs based on the plant components utilized, such as seeds, leaves, wood, or bark (53).

### Classification of phytogenics

#### Essential oils

The active ingredients in essential oils make them one of the most economically significant phytogenic compounds. Essential oils have been shown to play a significant role in protecting plants from insects, fungi, viruses, and bacteria (54–56). According to Grashorn (57), probiotics, particularly those belonging to the essential oil group, enhance the flavor and palatability of feed, which could enhance chicken performance and feed intake.

Essential oils (58), oleoresins (solvent-free), and natural extracts are phytochemicals that are generally recognized as safe (GRAS) for their intended use (59, 60). Essential oils are a variety of volatile oils extracted from plants; they possess an aroma and other distinguishing features of plants and are mostly employed in manufacturing perfumes, tastes, and medications (61).

#### Spices and herbs

Spices are strong or aromatic compounds of vegetable origin used as seasonings and preservatives, while herbs are flowering plants whose stems do not become woody and persistent and are prized for their flavor, fragrance, and medicinal qualities (61). Plant components such as roots, leaves, and bark are known as botanicals or phytobiotics, and they are utilized to create medications for therapeutic purposes.

**Figure 1:**
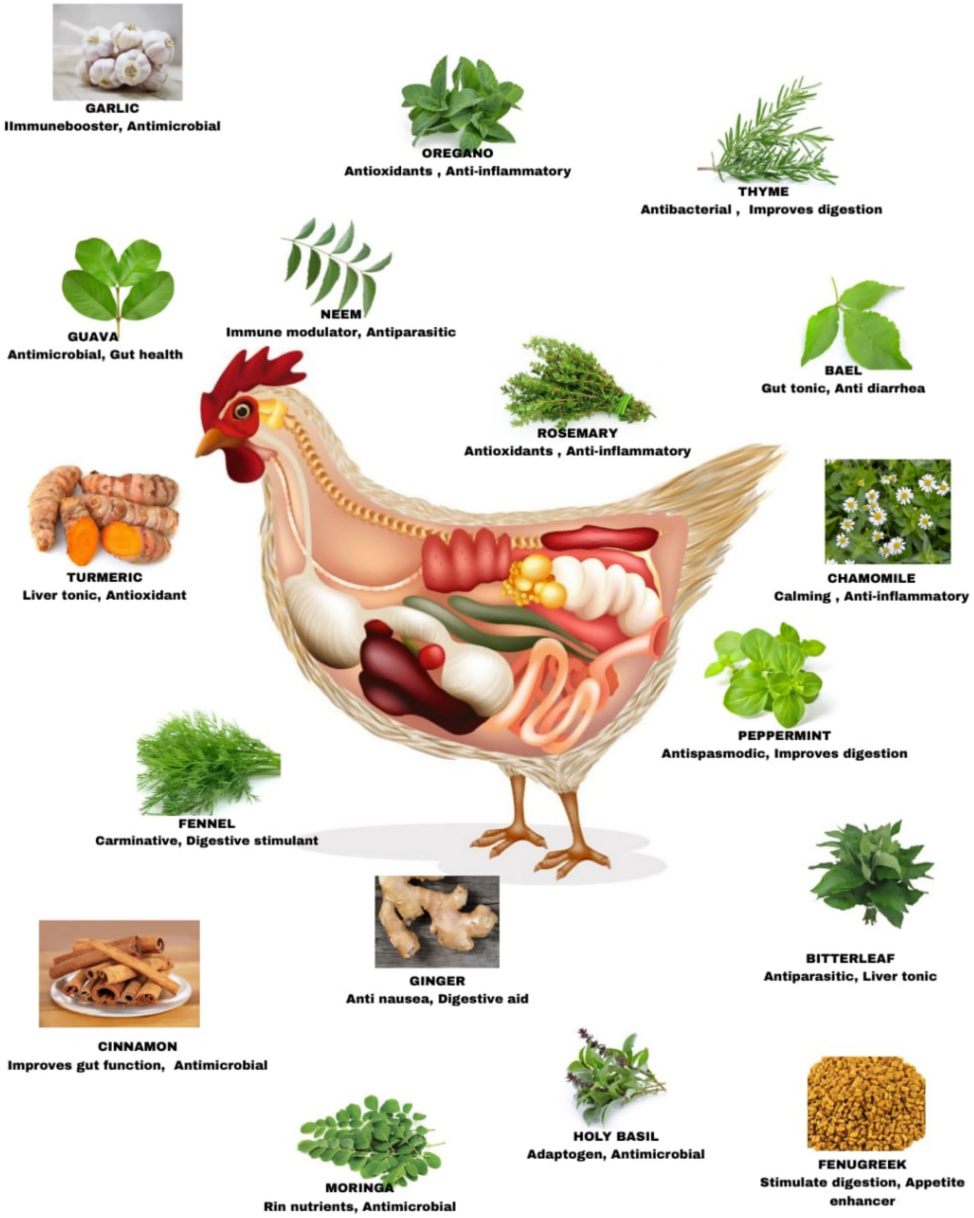
Common phytogenic additives and their functional roles in poultry gut health.

In chicken production, the most commonly used herbs and spices for phyto-feed additives include oregano, thyme, garlic, horseradish, chile, cayenne, pepper, peppermint, cinnamon, anise, clove, rosemary derivatives, citrus, and sage (62–65). A variety of phytogenic plants and their respective bioactive properties, which contribute to gut health modulation in poultry, are illustrated in Figure 1. Herbs such as turmeric, garlic, oregano, thyme, and neem exhibit antimicrobial, antioxidant, anti-inflammatory, and digestive-enhancing properties. These natural additives support intestinal integrity, immune function, nutrient utilization, and microbial balance, thereby offering sustainable alternatives to antibiotic growth promoters in poultry nutrition.

#### Effects of phytogenics on poultry

Several vitamins, minerals, and enzymes necessary for growth and development are found in phytogenics, which positively impacts the animal’s immunological response, growth performance, blood parameters, and carcass quality (66) (Figure 1). Diet, intestinal integrity, gut flora, and chicken immune system interact continuously to influence gut function and health, which directly impacts farm animal performance.

**Figure 2:**
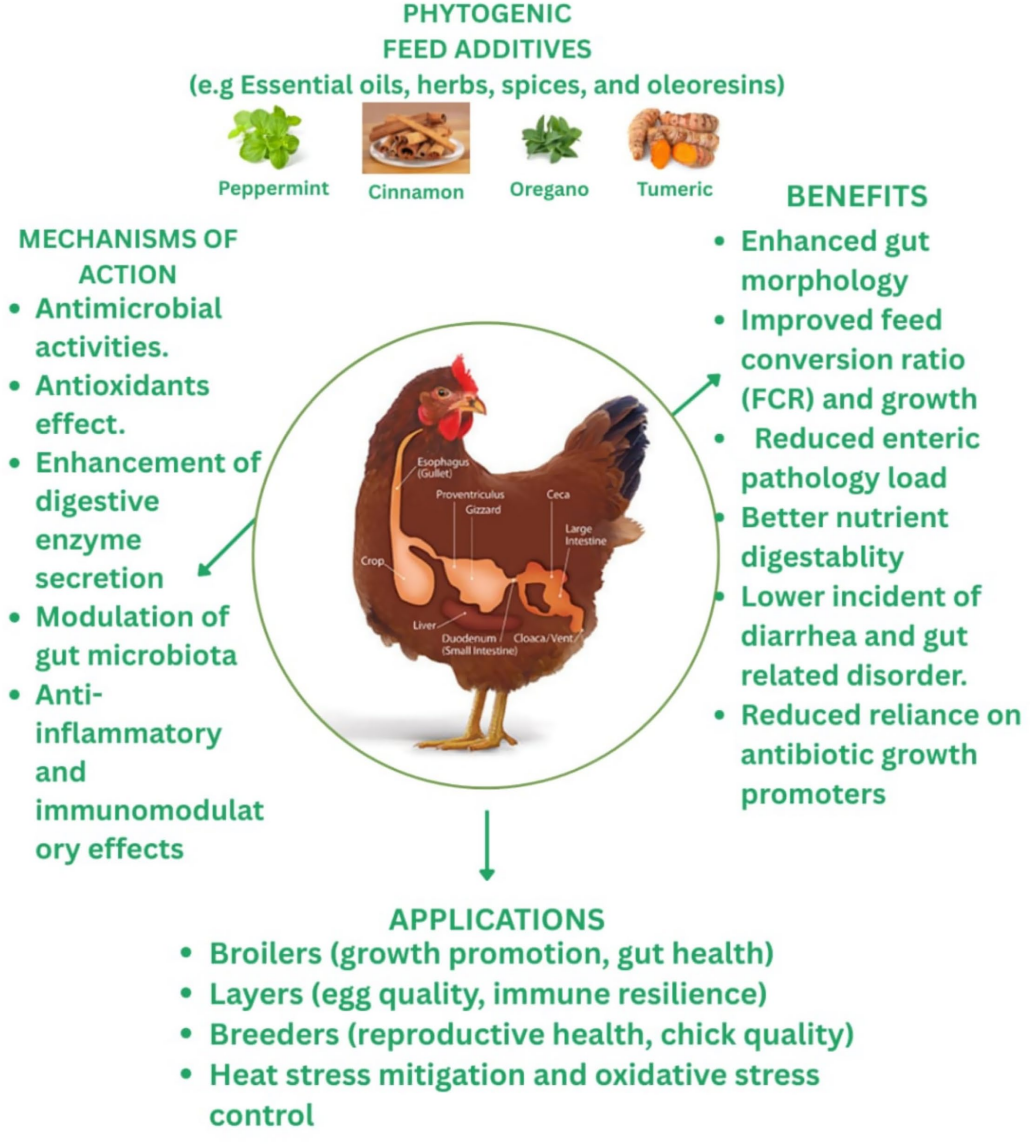
Gut health modulation through phytogenics in poultry.

Since phytogenic compounds contain a wide range of active components, they are among the most promising antibiotic substitutes. A better knowledge of how phytogenic compounds affect the three elements of the gastrointestinal ecosystem, gut microbiota, gut physiology, and immunology, as well as the mechanisms underlying these effects, may help us utilize phytogenic substances most effectively for sustainable and profitable animal production. The active ingredients in phytogenics include alkaloids, glycosides, tannins, and phenolic compounds (67). Through antimicrobial, antioxidant, and immunomodulatory mechanisms, phytogenic feed additives (PFAs), such as essential oils, herbs, and oleoresins, have several positive effects on the gut health of poultry (Figure 2). These activities improve performance traits, increase nutrient absorption, and create a favorable gut environment. Applications include egg-laying hens, breeders, broiler production, and stress reduction techniques, establishing PFAs as competitive substitutes for antibiotic growth promoters.

### Physiological and functional effects of phytogenics in the gut

Phytogenic feed additives (PFAs) exert their beneficial impacts on the gastrointestinal tract via various physiological mechanisms. They improve nutrient absorption and digestion by inducing the release of digestive enzymes. They increase feed efficiency (FE) by modifying the expression of hypothalamic neuropeptides linked to feeding behavior. Peripheral intermediate metabolisms, such as lipid metabolism and signaling pathways associated with protein synthesis, also regulate FE and are influenced by PFA bioactivities (43). PFAs enhance feed palatability, digestibility, and nutrient absorption. They also modulate the structure of the animal’s intestinal microbiome and enhance performance by promoting the biological activity of plant secondary compounds with antioxidative features. Additionally, PFAs inhibit the growth of pathogenic microorganisms in poultry (18). Certain phytogenics also strengthen gut morphology, boost immunological responses, and lessen the effects of environmental stressors, thereby contributing to improved poultry health and growth performance (19, 20, 62, 63). They have been shown to enhance gut health by decreasing pathogenic bacterial colonies, reducing fermentation by-products like ammonia and biogenic amines, lowering gut-associated lymphatic tissue activity, and improving the digestion of pre-caecal nutrients (43).

### Mechanisms of action of phytogenics on gut health

Phytogenics influence poultry gut health via some interrelated cellular, microbial, and molecular processes. Its antibacterial activity is one of its main modes of action. According to Yang et al. (18) and Yammine (68), compounds including thymol, carvacrol, and cinnamaldehyde reduce the growth of harmful bacteria like *Clostridium perfringens* and *Escherichia coli* by disrupting bacterial cell membranes, changing membrane permeability, and inhibiting quorum sensing. By specifically encouraging beneficial bacterial populations like Lactobacillus and Bifidobacterium, phytogenics not only have antibacterial qualities but also help to modulate the gut microbiota, supporting microbial balance and enhancing gut resilience (69, 70).

Because phytogenics contain secondary metabolites from plants, such as flavonoids and phenolic acids, they also have strong antioxidant properties. These substances protect the integrity of tight junctions and reduce inflammation by scavenging free radicals and reducing oxidative stress in intestinal epithelial cells (19, 20). Another crucial mechanism is immune modulation, whereby specific phytogenics enhance mucosal immunity and control the activity of gut-associated lymphoid tissue by activating innate immune responses via Toll-like receptor signaling and modifying the production of cytokines, such as interleukins and interferons (23, 71).

By promoting mucin production and upregulating the expression of tight junction proteins like occludin and claudin-1, phytogenics also improve intestinal barrier function by lowering intestinal permeability and preventing pathogen invasion (72, 73). Additionally, they encourage the release of digestive enzymes such as amylase and protease, which enhance the gut’s ability to absorb and digest food (23, 74). When taken as a whole, these processes highlight the diverse ways phytogenics support and enhance poultry intestinal health.

#### Influence of phytogenics on gut morphology and integrity

Phytogenic substances affect small intestine microflora by controlling potential pathogens. It increases the digestive system’s ability in the gastrointestinal tract, which may be an indirect side effect on the stability of gut microbiota. This increases the availability of essential nutrients in the intestine for absorption and helps to improve performance (75). According to Odunowo and Olumide (48) and Engida et al. (76, 77), PFAs’ active components boost chicken performance by promoting the production of digestive enzymes, which improves feed digestion and nutrient absorption and raises the feed conversion ratio in poultry (78). Additionally, by influencing physiological processes, including immune function and stress tolerance, they encourage animal ingestion, absorption, nutritional assimilation, and growth (79).

Additionally, the application of phytogenic compounds has been shown to help avoid digestive problems. According to Chrubasik et al. (80), a number of phytogenic compounds have demonstrated beneficial effects on the activity of digestive enzymes and gut microbiota. Mohamed and Hassan (15) also revealed that phytogenic compounds improve nutritional absorption and the activity of digestive enzymes. The gut environment is significantly impacted, either directly or indirectly, by phytogenic feed additives (81). It improves host immunity, food utilization, and intestinal histomorphology by acting as a broad-spectrum antibacterial across the digestive system (82, 83).

Vidanarachchi et al. (84) explained the mechanisms through which botanical powder and extract affect poultry. They concluded that botanical powder disrupts the cell membrane of microbes, affects the surface properties of microbial cells, enhances the growth of beneficial bacteria, enhances immune stimulants, encourages the growth of villus and crypt in the gut and stimulates the production and activity of the digestive enzymes. Also, Abdel-Moneim et al. (85) and Prihambodo et al. (86) demonstrated that flavonoids in herbs possess a beneficial impact on the gut of poultry. They showed that flavonoids can improve the small intestine’s ability to absorb nutrients and have antioxidant qualities. Additionally, other phytogenic substances, including genistein and hesperidin improved gut morphology, such as villus height, crypt depth, and density (87). Then, deeper crypts encourage quick villi regeneration in response to inflammation caused by pathogens, whereas higher villi expand the intestinal surface area and enhance food absorption (88).

The potential of phytogenic bioactive compounds to stimulate the proliferation and growth of absorptive cells in the gastrointestinal tract (resulting in greater villus height and deeper crypt) (89), and to influence the production and/or activity of the digestive enzymes, e.g., increasing the activities of amylase and protease (90, 91), have also been thought to improve the growth performance of birds.

By raising the amounts of Bacteroides spp., Clostridium cluster IV, and Clostridium cluster XIVa associated with the cecal mucosa, it was demonstrated that supplementing a PFA with carvacrol as the primary active ingredient would alter the intestinal microbiota more at the cecal than the ileal level (92). Given that Clostridium is not only predominant in the ceca (93) but also helps maintain the general health of the gut, particularly through the generation of butyrate (94), this rise may be advantageous. Ahsan et al. (95) found that the addition of a mix of essential oils to the broiler diet significantly improved gut villus height and width and decreased crypt depth. According to Navarro et al. (96), broiler chickens fed an equal blend of carvacrol and thymol as a feed supplement showed improved growth-promoting effects on immunological response, fatty acid composition, antioxidant enzyme activities, and performance. Furthermore, it has been noted that extracts from oregano and other herbs can inhibit the formation of dangerous coliform bacteria in grill chickens while leaving good bacteria unaffected (97).

Steiner (98) reported that improving nutrient availability in broiler diets with the addition of phytogenic products may improve villus length. Adibmoradi et al. (99) showed linearly enhanced villus height and gut crypt depth with garlic inclusion in broiler feed from 0.125 to 2%. Also, Oladele et al. (100) studied the effect of garlic meal at 0.125, 0.25, and 0.5% levels on the absorptive surface of the small intestine of broilers. They reported that 0.125% garlic meal supplementation improved villus length, width and cryptal depth, resulting in an increased absorptive area of the intestine and improved performance. Saeid et al. (101) concluded that intestinal morphological characteristics (villi length and small crypt) improved in broiler feeding a 0.5% garlic powder-containing diet compared to the control. The improvement in performance may be because garlic has positive effects on gut flora by reducing pathogenic bacteria. The immune promotion was considered as pronouncing action through that phytogenic have beneficial impacts on gut health (102). Karangiya et al. (103) concluded that supplementation of ginger to broiler diet at 1% significantly (*p* < 0.05) increased villi length, width and cryptal depth, indicating increased absorptive surface area.

Microflora activity in the gut is influenced by the role of poultry microbiota and the stabilizing effects of phytogenic chemicals on the gut environment (104). In 2013, Vukić-Vranješ et al. (105) concluded that additional phytogenic additives improved broiler performance, measured jejunum shape, and decreased the quantity of harmful bacteria. Mohamed and Hassan (15) revealed that botanical powder could break down the microbial cell membrane, alter the surface characteristics of microbial cells, promote the growth of good bacteria, boosts immunological stimulants, promote the development of crypt and villus in the gut, and increases the synthesis and activity of digestive enzymes.

#### Phytogenic additives and gut-associated immune response

In poultry farming, gut health has been the focus of intensive research (11). It has been described as the “state of symbiotic equilibrium between the intestinal tract and microbiota where animal health and welfare remain unaffected.” It is considered of utmost importance (106). A sizable percentage of immune cells are found in the gut, making it an important part of the immune system. In order to provide defence against pathogens and lower the risk of infections such as enteric illnesses, a healthy gut microbiota helps to promote mucosal immunity.

Conway (14) suggested that the mucosa, commensal bacteria, and diet are the three variables that makeup gut health. Gut-associated lymphoid tissue (GALT), the mucus covering the digestive epithelium, and the digestive epithelium itself, with its unique shape, make up the mucosa. The GALT, mucus layer, microbiota and host epithelium relate to form a complex and unique equilibrium within the GIT that brings about an effective working of the digestive system. Consequently, it was suggested that gut health be defined as “a stable state where the intestinal tract and microbiome coexist in symbiotic equilibrium and where intestinal dysfunction does not limit the performance and welfare of the animal (107).

#### Mechanisms of action of phytogenic additives in gut health modulation

As natural feed additives, phytogenics are being explored extensively in animal nutrition to improve animal performance, health, and overall productivity (108). Due to their numerous advantages, phytogenic feed additives (PFA) are becoming more and more important in animal nutrition and as possible alternatives to antibiotics. They are known to exhibit immunomodulatory, antioxidant, and antimicrobial properties, which help to improve growth, enhance nutrient utilization efficiency, modulate gut health and functions, and lessen the adverse environmental effects of poultry production (109).

#### Antimicrobial effects of phytogenic compounds

The antimicrobial properties help lower the number of intestinal pathogens by suppressing adhesion in the mucosa. Its major objective is to improve gut health, increasing the capacity for absorption and digestion (15). Antimicrobial substances have primarily targeted the biosynthetic machinery of bacterial cell walls (110). The cytoplasm and cell walls, including membranes, can be affected by EOs and their derivatives, which frequently results in a radical alteration of the cell’s shape. These characteristics make EOs a viable source of phytogenics (18). EOs cause bacterial cell membranes to become more permeable, allowing cell contents to seep and ultimately killing the cell.

The phenolic compounds found in thyme, oregano, and clove essential oils, such as thymol, carvacrol, and eugenol, give them antibacterial properties. The antimicrobial properties of phenolic components may be defined as interactions between the microbial cell membrane and viable materials, which are then linked to the hydrophobicity of these materials and have the potential to harm the cytoplasm and cell wall (111). Koscova et al. (112) showed that employing a combination of carvacrol and thymol may effectively combat *C. perfrigens* in broiler guts and lessen the impact of necrotic enteritis outbreaks.

Additionally, thymol, cinnamon aldehyde, and carvacrol had a minor impact on *E. coli* bacteria but a significant effect on *Salmonella enterica* (113). Numerous active ingredients in neem oil, according to Ansari et al. (114), stimulate lymphocytes and macrophages to improve the immunological response. Khan et al. (115) found that garlic, oregano, and thyme had a significant effect on a variety of bacteria. A combination of carvacrol and thymol or 1% carvacrol oil supplementation dramatically decreased campylobacter levels, according to Arsi et al. (116). According to Singh et al. (117) and Alagbe (118), phytochemical compounds like flavonoids, phenols, and alkaloids can act as probiotics by using competitive exclusion to decrease the activity of harmful bacteria and encouraging the growth of good bacteria like *Lactobacillus* sp.

#### Anti-inflammation effect of photogenic compounds

Gut development and the effectiveness of nutrient consumption are negatively impacted by gut inflammation. Numerous studies have shown that alterations in gut architecture, mucosa damage, increased mucosal permeability, poor gut growth, and decreased capacity to absorb nutrients are all linked to gut inflammation and chronic inflammatory disorders (18, 119). In general, there are three categories of intestinal inflammation: weaning-associated, diet allergy-associated, and infection-associated. Even while the inflammation does not elicit the entire range of clinical symptoms, it significantly impairs performance and results in financial losses.

The immune system’s “watch dogs” are gut epithelial cells. By using cytokines that are essential for the acquisition and activation of neutrophils, macrophages, T and B cells, and dendritic cells, they can indicate the start of the host’s innate and acquired immune responses or inflammation (18). According to a recent study, cinnamon oil supplementation reduced inflammation and LPS-induced damage (120). Also, capsicum, cinnamon aldehyde, and carvacrol—at low concentrations had immune-boosting qualities that might shield broiler chickens from infection with the live coccidiosis challenge (121).

#### Phytogenic substances as antioxidants

Antioxidant properties are a common characteristic of many PFA, while the specific effects can differ depending on the kind of plant, its origin, extraction methods, and dietary makeup (19). Phytochemicals are capable of changing the antioxidant capacity of poultry by changing the meat’s fatty acid composition from saturated (lauric, myristic, palmitic, and stearic acids) to monounsaturated (like oleic acid) and polyunsaturated (PUFA) (long-chain) fatty acid lineages (52).

It was reported by Wei and Shibamoto (122) that phytogenic compounds had antioxidant capabilities. The majority of the antioxidant properties of phytogenics are attributed to terpene and phenolic components, including rosmarinic acid. Furthermore, several phytogenic compounds, such as thyme, oregano, green tea, and pepper, that contain flavonoids, carvacrol, and thymol, also function as antioxidants (123). PFA protects birds from the harmful effects of lipid peroxidation by enhancing their antioxidant capabilities, as numerous studies have shown (78, 124). There is evidence that PFA can support the gut lining as an antioxidant, balancing the production of free radicals and their neutralization (125).

According to Botsoglou et al. (126), feeding turkey oregano extract as a supplement considerably reduced the amount of fat oxidation in the flesh. Therefore, phytogenic compounds may enhance the quality of poultry products’ flesh. In comparison to commercial antioxidants like ascorbic acid and vitamin E, Luna et al. (127) discovered that adding carvacrol or thymol to chicken diets dramatically decreased lipid oxidation. Consequently, there are no negative performance implications when using phytogenic compounds as an antioxidant in the diet of animals and poultry. High antioxidant concentrations in phytogenic compounds may change the peroxidation state and antioxidant capability beneficially (51, 128). Furthermore, using phytogenics as feed additives may help mitigate the effects of heat stress because of their high antioxidant content (66, 129).

#### Phytogenic substances as an antivirals

Phytogenics are useful in disease prevention and control methods to improve the immune system’s ability to fight off disease and prevent problems with sickness (130). Numerous phytogenic compounds have antiviral effects (Table 1). Newcastle virus growth in embryos may be inhibited by using *Artemisia annua* (29). A combination of essential oils added to the broiler feed improved the immune responses against the H5N1 and H9N2 vaccines, according to research by Barbour et al. (131) and Jiang et al. (132). Additionally, Lee et al. (124) found that the H9N2 virus’s development was inhibited when green tea extract was added to broiler feed or water. According to El-Shall et al. (133), adding a blend of essential oils that included oregano, carvacrol, thyme, eucalyptus, thymol, eucalyptol, and acacia surfactant to broiler chickens’ drinking water stimulated their immune systems against infectious bursal disease and Newcastle disease (ND) vaccines, as well as having an antiviral effect against the ND virus.

**Table 1:** Essential oil-based phytogenics.

Phytogenic	Active compounds	Dosage	Gut health effects	References
Oregano	Thymol, Carvacrol, Eugenol	20 mg/kg	Increased villi height; reduced crypt depth in jejunum; stimulates beneficial microbiota (Lactobacillus, Bifidobacterium)	Zhang et al. (51) and Madkour et al. (143)
Cinnamon	Cinnamaldehyde, Eugenol	–	Reduces pathogenic bacteria (*E. coli*, Campylobacter); promotes Lactobacillus and Bifidobacterium	Zeng et al. (144), Devi et al. (145), and Rashid et al. (146)
Thyme	Thymol, Linalool, Carvacrol	–	Enhances digestive enzyme production; promotes beneficial microflora	Vlaicu et al. (147) and Soliman et al. (148)
Rosemary	Essential oils, phenolic acids, flavonoids	10 g	Enhances gut-beneficial microbial composition; reduces pathogens	Engida et al. (76, 77)

### Phytogenics and their effects on gut health

Tables 1–4 categorize phytogenic compounds based on their primary bioactive components and physiological effects on poultry gut health. Table 1 highlights essential oil–based herbs such as oregano, cinnamon, thyme, and rosemary. Their active constituents, such as thymol, carvacrol, cinnamaldehyde, and linalool exert antimicrobial effects, stimulate beneficial gut microbiota (e.g., Lactobacillus, Bifidobacterium), and improve intestinal morphology by increasing villus height and enzyme secretion. These phytogenics are widely recognized for enhancing digestive efficiency and protecting gut health and integrity. The herbal extracts with anti-inflammatory or antioxidant properties, such as turmeric, black cumin, and bitter leaves, are shown in Table 2. Rich in bioactives like curcumin, thymoquinone, and flavonoids, these herbs reduce intestinal inflammation and oxidative stress, support mucosal healing, and enhance intestinal morphology. Their protective roles make them particularly valuable in poultry that is stressed or exposed to pathogens. Plants such as chicory and moringa are presented in Table 3 due to their high content of fermentable fibres and polyphenols. These compounds act as prebiotics, enhancing the growth of beneficial gut microbes and improving intestinal architecture. Their inclusion in diets has been linked to enhanced villus development and the inhibition of enteric pathogens, such as *E. coli* and Salmonella. This diverse group (Table 4) includes sweet wormwood, garlic, and ginger. These herbs enhance gut health through mechanisms such as boosting digestive enzyme secretion, improving motility, and strengthening the gut barrier. Their bioactive compounds (e.g., artemisinin, allicin, gingerol) also exhibit antimicrobial activity and aid in nutrient absorption.

**Table 2:** Herbal extracts with anti-inflammatory or antioxidant properties.

Phytogenic	Active compounds	Dosage	Gut health effects	References
Turmeric	Curcumin	0.5–1%	Reduces gut inflammation and oxidative stress; prevents leaky gut; inhibits harmful bacteria	Laganá et al. (149), Yadav et al. (150), Oke (151), Kpomasse et al. (152), and Onagbesan et al. (153)
Black Cumin	Thymoquinone	2–3%	Reduces gut inflammation; improves intestinal integrity and gut morphology	Seidavi et al. (154), Oke et al. (155), and Oyelola et al. (156)
Bitter Leaf	Flavonoids	–	Improves intestinal mucosa’s digestive enzymes and absorption capacity	Tokofai et al. (157–159)

**Table 3:** Prebiotic or fiber-rich plants.

Phytogenic	Active compounds	Dosage	Gut health effects	References
Chicory	Inulin	1–1.5%	Acts as a substrate for beneficial gut microorganisms; increases gut morphometry parameters	Gurram et al. (160)
Moringa	Quercetin, Flavonoids, Polyphenols, Glucosinolates	–	Increases villus surface area; promotes beneficial bacteria; inhibits Salmonella and *E. coli*	Soundararajan et al. (161) and Gul et al. (162)

**Table 4:** Other herbal phytogenics.

Phytogenic	Active compounds	Dosage	Gut health effects	References
Sweet Wormwood	Artemisinin	1%	Enhances digestive enzyme activity; promotes gastric motility; reduces gut inflammation	Cui et al. (163)
Garlic	Allicin, Alliin, Diallyl Disulfide	0.25–0.75 g/kg, 1%	Enhances pancreatic enzyme activity; lowers gut pH; improves nutrient absorption and gut barrier function	Karangiya et al. (103), Ismail et al. (164), Abd El-Ghany (165), Adjei-Mensah et al. (166)
Ginger	Gingerol, Zingiberene, Shogaol	1–1.5%	Enhances gastric secretion; improves digestion and nutrient absorption; reduces *E. coli* and Salmonella	Dieumou and Teguia (167) and Dosu et al. (168)

### Challenges and prospects for phytogenic supplementation in poultry diets

Although the potential benefits of phytogenic feed additives, ranging from enhanced gut health and immunity to improved growth performance, are well documented. However, their widespread adoption remains limited due to several practical and scientific challenges. A significant constraint is the variability in the composition and effectiveness of phytogenic products. These inconsistencies stem from biological factors such as plant species, cultivation location, harvest timing, and processing techniques like extraction or distillation. Additionally, storage conditions (light, temperature, oxygen exposure, and duration) can influence the stability and bioactivity of phytogenic compounds (67, 134, 135). This variability undermines confidence among producers and complicates the standardization of phytogenic products (19–21).

Furthermore, phytochemicals exhibit dose-dependent effects, with research indicating that more pronounced benefits are observed when administered during early life stages in poultry (136). However, suboptimal or inconsistent dosing can lead to ineffective outcomes (137). Some phytogenic substances may also produce undesirable effects such as off-flavors, toxicity, or interactions with other feed ingredients (138, 139). The volatility of certain compounds further contributes to inconsistent concentrations in final products (18). Economic factors also present barriers. The relatively high cost of many commercial phytogenic additives, coupled with limited field-scale trials that validate their benefits in commercial settings, makes their adoption less attractive, especially in low-and middle-income countries (109). Moreover, a general lack of technical knowledge among farmers and feed manufacturers hinders proper application and potential impact.

Despite these limitations, several emerging trends and innovations offer promise for the broader adoption of phytogenic products. The global shift toward antibiotic-free (ABF) poultry production creates a conducive environment for the use of natural additives. Phytogenics, with their demonstrated roles in pathogen control, immune modulation, and gut health, are well suited for ABF systems (62, 63, 140, 141). Advances in formulation technology, such as microencapsulation and standardized extraction techniques, are enhancing product consistency and bioavailability (21, 142). Additionally, the increased availability of cost–benefit analyses linking phytogenic use to improved feed conversion, growth, and health outcomes will strengthen their economic justification. Supportive regulatory frameworks, along with livestock farmer training, extension services, and on-farm demonstrations, will also play an important role in integrating phytogenics into mainstream poultry nutrition.

### Future perspectives and directions

The application of phytogenic feed additives in poultry production is a promising alternative to antibiotic growth promoters, yet several critical knowledge gaps hinder their optimal use. Future research should first focus on standardizing phytogenic formulations, particularly through quantitative profiling and identification of bioactive constituents using advanced analytical tools, such as LC–MS/MS and NMR spectroscopy. This will help to reduce variability in efficacy across batches and production systems. Secondly, precise dose–response studies are needed to establish minimum effective concentrations, identify potential toxicity thresholds, and evaluate their interactions with feed matrices under commercial pelleting and extrusion processes. Research into the stability of compounds during storage and feed manufacturing is also required to enhance product shelf life and ensure consistent performance.

Another priority is the investigation of synergistic or antagonistic interactions between phytogenics and other functional feed additives (like probiotics, prebiotics, enzymes, and organic acids). For example, studies should explore whether combinations enhance gut barrier integrity or modulate immune responses more effectively than when used alone, using factorial design experiments and host-microbiome readouts. The development and testing of advanced delivery technologies, such as microencapsulation, nanoemulsions, and pH-sensitive coatings, can enhance the stability, bioavailability, and site-specific release of phytogenic compounds in the gastrointestinal tract. These technologies should be evaluated through controlled-release assays and gut transit studies using markers or imaging. To unravel the precise mechanisms of action, integrating multi-omics platforms (such as transcriptomics, proteomics, metabolomics, and microbiomics) is crucial. These approaches can provide insights into host-gut microbiota interactions, immune modulation, and metabolic shifts induced by specific phytogenic compounds or their combinations. Moreover, longitudinal *in vivo* trials across diverse poultry genotypes, production systems (such as intensive versus free-range), and environmental conditions are necessary to assess the robustness, reproducibility, and long-term effects of phytogenic supplementation on productivity, welfare, and product quality.

Furthermore, the establishment of regulatory frameworks, safety thresholds, and validated quality control protocols is vital for the large-scale commercialization of phytogenics. Such frameworks should be based on sound scientific data and harmonized across regions to ensure the safe, sustainable, and economically viable integration of phytogenics into poultry nutrition programs, particularly in the context of antibiotic-free production and climate-resilient livestock systems.

## Conclusion

A viable and successful method for enhancing gut health and general performance in poultry production is using phytogenic feed additives. By strengthening immune responses, improving nutritional absorption, and improving gut microbial balance, their varied bioactive components help to lessen the need for antibiotics. Despite the well-established advantages of phytogenics, issues including compositional variability, standardization, and cost-effectiveness need to be resolved. Future studies should focus on improving their use through creative delivery methods, optimized formulations, and thorough assessments of their long-term effects. The industry can increase animal sustainability, productivity, and health by incorporating phytogenics into chicken feed, opening the door to poultry production systems that do not use antibiotics.
